# Radiomics and deep learning methods for predicting the growth of subsolid nodules based on CT images

**DOI:** 10.1097/MD.0000000000044104

**Published:** 2025-08-29

**Authors:** Jie Chen, Wanying Yan, Yiqiu Shi, Xinyu Pan, Ruize Yu, Dawei Wang, Xinyue Zhang, Lina Wang, Kefu Liu

**Affiliations:** a Department of Radiology, The Affiliated Suzhou Hospital of Nanjing Medical University, Suzhou, Jiangsu, China; b Infervision Medical Technology Co., Ltd, Beijing, China; c Department of Medical Imaging, The Affiliated Suqian First People’s Hospital of Nanjing Medical University, Suqian, Jiangsu, China; d Department of Epidemiology & Biostatistics, School of Public Health, Southeast University, Nanjing, Jiangsu, China.

**Keywords:** computed tomography, deep learning, growth, radiomics, subsolid nodule

## Abstract

The growth of subsolid nodules (SSNs) is a strong predictor of lung adenocarcinoma. However, the heterogeneity in the biological behavior of SSNs poses significant challenges for clinical management. This study aimed to evaluate the clinical utility of deep learning and radiomics approaches in predicting SSN growth based on computed tomography (CT) images. A total of 353 patients with 387 SSNs were enrolled in this retrospective study. All cases were divided into growth (*n* = 195) and non-growth (*n* = 192) groups and were randomly assigned to the training (*n* = 247), validation (*n* = 62), and test sets (*n* = 78) in a ratio of 3:1:1. We obtained 1454 radiomics features from each volumetric region of interest (VOI). Pearson correlation coefficient and the least absolute shrinkage and selection operator (LASSO) methods were used for radiomics signature determination. A ResNet18 architecture was used to construct the deep-learning model. The 2 models were combined via a ResNet-based fusion network to construct an ensemble model. The area under the curve (AUC) was plotted and decision curve analysis (DCA) was performed to determine the clinical performance of the 3 models. The combined model (AUC = 0.926, 95% CI: 0.869–0.977) outperformed the radiomics (AUC = 0.894, 95% CI: 0.808–0.957) and deep-learning models (AUC = 0.802, 95% CI: 0.695–0.899) in the test set. The DeLong test results showed a statistically significant difference between the combined model and the deep-learning model (*P* = .012), supporting the clinical value of DCA. This study demonstrates that integrating radiomics with deep learning offers promising potential for the preoperative prediction of SSN growth.

## 1. Introduction

Owing to the widespread use of low-dose computed tomography (CT) in lung cancer screening, the detection rate of subsolid nodules (SSNs) has significantly increased. Persistent SSNs exhibit heterogeneous growth patterns.^[1]^ While most lesions follow an indolent course, some demonstrate rapid progression within a relatively short period,^[2–4]^ often characterized by an increase in nodule size and the appearance or enlargement of intralesional solid components.^[5,6]^ Given this variability, SSN growth is considered a key prognostic marker for malignancy and plays a crucial role in predicting the clinical course of lung disease. However, in clinical practice, long-term CT surveillance can lead to patient anxiety, increased healthcare costs, and cumulative radiation exposure, potentially contributing to overdiagnosis. Therefore, a reliable tool for predicting SSN growth is essential to optimize patient management and minimize the risk of overtreatment.

Previous studies have explored the predictive value of SSN growth using conventional CT-based quantitative parameters, such as nodule diameter and mean CT attenuation.^[5–8]^ However, their findings are limited by small sample sizes, inconsistent follow-up intervals, and variations in image reconstruction techniques, which compromise the reliability of the results.^[9,10]^

Recent advances in radiomics have enabled more individualized assessment of SSNs. By transforming digital medical images into high-dimensional quantitative features, radiomics facilitates noninvasive and personalized clinical decision-making.^[11,12]^ Several studies have demonstrated the potential of radiomics in predicting SSN growth patterns.^[13,14]^ Nonetheless, its application in dynamically monitoring nodules and predicting interval growth remains challenging due to limited case numbers and the requirement for extended follow-up durations.

Deep learning has emerged as a cutting-edge tool for the segmentation and classification of medical imaging data. It transforms low-level representations into higher-level, more abstract features by combining simple yet nonlinear modules. Compared with radiomics, deep learning reduces the manual labeling burden on physicians and enables automatic extraction of high-order imaging features. To the best of our knowledge, only a few studies^[15,16]^ explored the use of deep learning for predicting the growth of SSNs.

Therefore, we aimed to develop a predictive approach that integrates radiomics and deep learning to support clinical decision-making regarding the follow-up and treatment of patients with SSNs.

## 2. Materials and methods

### 2.1. Patients

The study protocol was approved by the Medical Ethics Committee of the Affiliated Suzhou Hospital of Nanjing Medical University (approval number: K-2021-GSKY20210209), and the requirement for informed consent was waived. From January 2017 to October 2022, 2358 patients with 3120 SSNs were retrospectively reviewed and analyzed at our institution. All eligible SSNs were incidentally detected during routine the annual CT examinations.

The inclusion criteria were as follows: patients who underwent non-contrast, thin-section CT scans; a CT follow-up period of ≥3 years for the target SSN, or <3 years if the SSN exhibited growth during the follow-up; SSNs that persisted during follow-up and were not resected within the first 2 years; SSNs with an initial maximum diameter between 6 mm and 30 mm on baseline CT; and availability of complete clinical data.

The exclusion criteria were as follows: SSNs that partially or completely resolved during follow-up; patients who underwent only a single CT examination; patients with a follow-up duration of <3 years without evidence of growth; and history of malignancy within the past 5 years; or CT images with obvious artifacts around the nodules.

The nodules were divided into growth and non-growth groups. A growing SSN was defined as an increase in the in diameter, an enlargement of the solid component to ≥2 mm, or the appearance of a new intralesional solid portion.^[17]^ A non-growing SSN was defined as one that remained stable in size and attenuation throughout the follow-up period.

**Figure 1.**
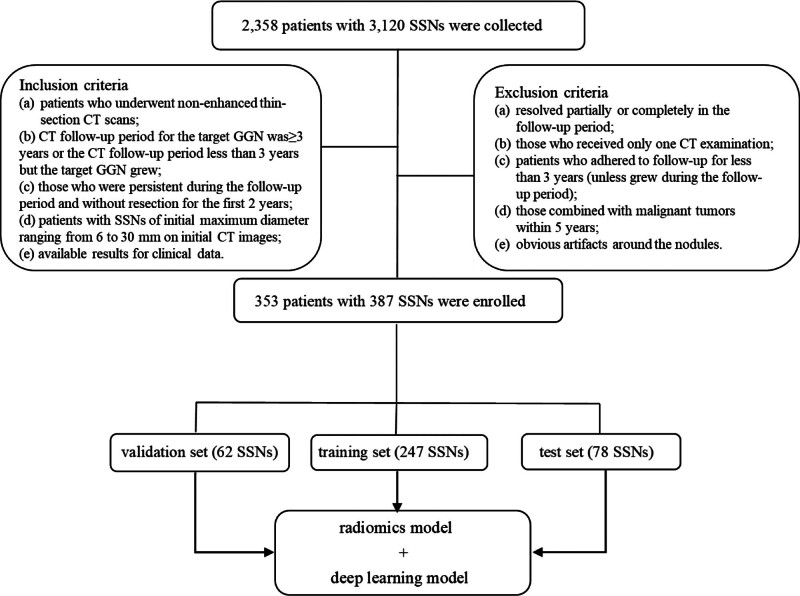
Flowchart of case enrollment and dataset assignment.

The total observation period was defined as the interval between the initial and final CT scans for the same SSN, or between the initial CT scan and the time of the last intervention. Ultimately, 353 patients with 387 SSNs met the inclusion criteria and were enrolled in this study. Among these, 192 SSNs were assigned to the growth group and 195 SSNs to the non-growth group. The patients were randomly divided into a training set (247 SSNs), validation set (62 SSNs), and test set (78 SSNs) in a ratio of 3:1:1. A flowchart of case selection is presented in Figure 1.

### 2.2. CT scanning

All images were obtained using a 64-slice CT scanner (SOMATOM Perspective, Siemens, Germany). The imaging parameters were as follows: tube voltage, 100 to 120 kVp; automatic tube current modulation; collimation, 0.6 mm × 64 mm; matrix, 512 × 512; rotation time, 0.37 seconds; and reconstruction slice thickness of 0.75 mm with a 0.5 mm reconstruction interval.

### 2.3. Image interpretation by radiologists

The SSNs were assessed by 2 radiologists (JC and YS, with 6 and 3 years of experience in thoracic imaging, respectively) based on thin-section, non-contrast CT images. In cases of disagreement, a third radiologist (KL, with 20 years of thoracic imaging experience) served as the arbitrator. All CT images were uploaded in DICOM format to the InferScholar multimodal scientific research platform (https://research.infervision.com/v2/) for image annotation. Segmentation was performed by radiologist JC. The boundaries of the volumetric region of interest (VOI) were manually adjusted slice by slice, if necessary, to exclude normal vessels and adjacent nodules, or in case where the nodule margins were ill-defined.

**Figure 2.**
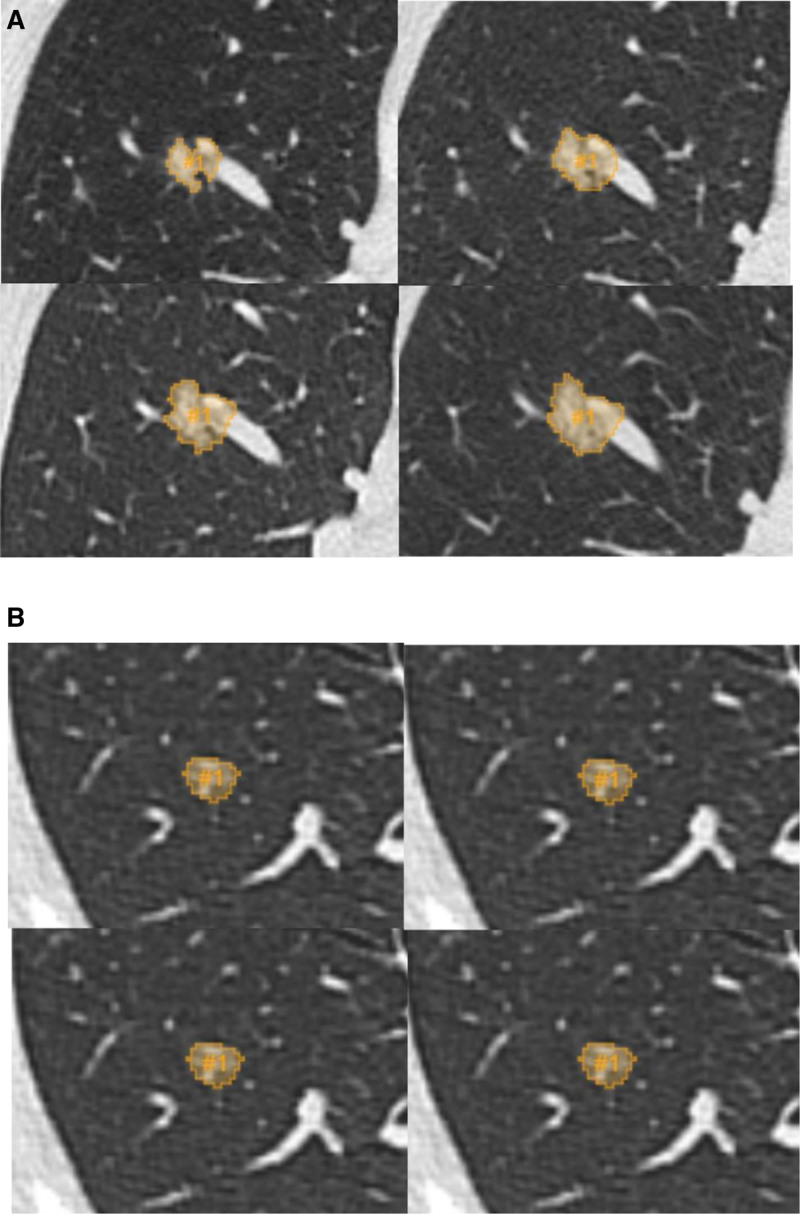
Representative CT segmentation images of ROIs for (A) growing and (B) non-growing subsolid nodule during preoperative follow-up. CT = computed tomography, ROI = region of interest.

To ensure segmentation accuracy, a second review of all segmented lesions was conducted by radiologist KL. Representative examples of lesion segmentations are presented in Figure 2.

### 2.4. Radiomics feature extraction

**Figure 3.**
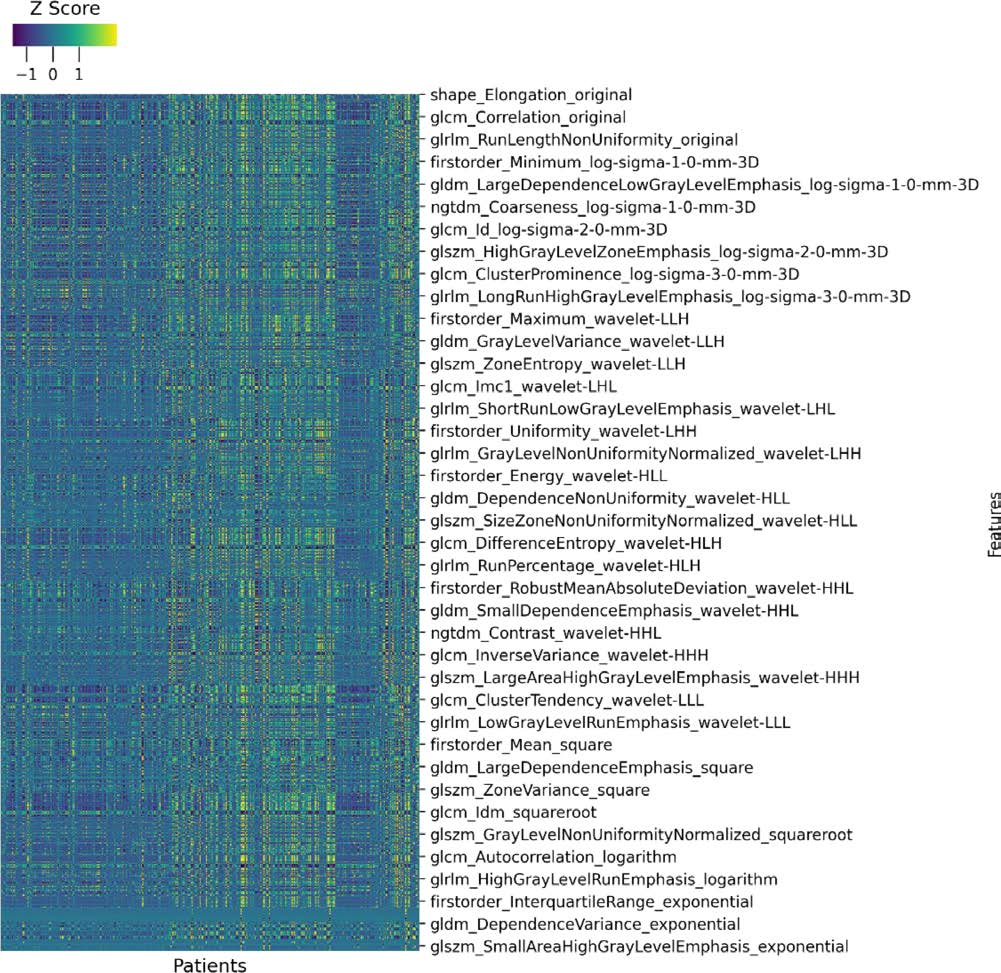
Heat map of retained radiomic features after ICC-based stability filtering. ICC = intra-class correlation coefficient.

Radiomics features were extracted using the InferScholar platform, resulting in 1454 raw features, including 288 first-order features, 336 gray-level co-occurrence matrix (GLCM) features, 224 gray-level dependence matrix (GLDM) features, 256 gray-level run-length matrix features, 256 gray-level size zone matrix (GLSZM) features, 80 neighborhood gray-tone difference matrix (NGTDM) features, and 14 shape features. To assess the reproducibility of radiomics feature extraction, 60 patients were randomly selected 3 months later. Intra-class correlation coefficients (ICCs) were calculated to evaluate the consistency of the extracted features.^[18]^ Only features with both inter- and intra-observer ICCs > 0.75 were retained. A heat map of the remained radiomic features is presented in Figure 3. All extracted features were normalized using Z-score preprocessing.

### 2.5. Radiomics model building and validation

Given the large number of retained features, a sequential feature selection pipeline was implemented to reduce redundancy and enhance model robustness. Pearson correlation coefficients were calculated, and features with correlation values >0.9 were removed. Subsequently, the least absolute shrinkage and selection operator (LASSO) regression was applied to further identify the most predictive features.

This multi-step selection process resulted in 30 final features, which were used to construct the radiomics model (see Supplemental Content 1 [Supplemental Digital Content, https://links.lww.com/MD/P754], which illustrates the radiomics features retained after feature selection).

Based on these features, 7 machine-learning models were developed: XGBoost, logistic regression, LightGBM, k-nearest neighbor, Naïve Bayes, support vector machine, and random forest. Three-fold cross-validation was performed during training to optimize hyperparameters and improve model performance.

### 2.6. Deep-learning model

A deep-learning model was developed for the classification task using the ResNet18 architecture. Image normalization was applied to ensure consistent intensity distribution across all samples. The VOI region was determined based on physician-delineated segmentation, effectively eliminating irrelevant background areas. The region of interest (ROI) area was calculated in each annotated slice. To preserve spatial context, the slices immediately above and below the one containing the largest ROI were retained, forming a 3-channel input image for each CT scan. During model training, data augmentation techniques – including random rotation, horizontal flipping, shifting, and scaling – were applied to mitigate overfitting. The preprocessed images were then passed through the convolutional layers of ResNet18 to extract deep learning features. To enhance interpretability, Gradient-weighted class activation mapping (Grad-CAM) was applied to the trained ResNet18 network. The resulting heatmaps were overlaid on the original CT images to visualize the regions that contributed most to the model’s predictions. Representative slices with well-defined lesion boundaries and surrounding context were selected for display.

### 2.7. Combined radiomics and deep-learning model construction

**Figure 4.**
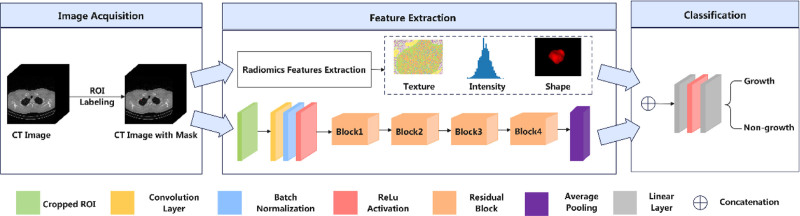
Flow chart of combined analysis for predicting growth of subsolid nodules.

To leverage the complementary strengths of radiomics and deep learning, a ResNet-based fusion network was constructed during the feature extraction phase. The backbone architecture of the fusion model was adapted from ResNet18, with the same preprocessing procedures applied as in the standalone deep-learning model. Following the residual blocks and the average pooling layer, radiomics features previously filtered based on ICCs were concatenated with the deep learning features. The fused feature vector was then passed through fully connected layers and a Softmax activation function to produce the final binary classification probabilities. The complete architecture of the fusion model is illustrated in Figure 4.

### 2.8. Statistical analysis

Statistical analyses were performed using SPSS 22.0. Quantitative data were presented as mean ± standard deviation, while qualitative data were expressed as percentages. Independent-sample *t*-tests and Mann–Whitney *U* tests were performed to compare the differences in continuous variables. Categorical variables were compared using the chi-square test. Model performance metrics – including accuracy, sensitivity, and specificity – were calculated for each model. Receiver operating characteristic (ROC) curve analysis was conducted to evaluate the diagnostic performance, and the area under the curve (AUC) was calculated. AUC values were compared between cohorts using the DeLong test. Model calibration was assessed using the Hosmer–Lemeshow goodness-of-fit test. Decision curve analysis (DCA) was performed to evaluate the clinical utility of the models. A 2-sided *P*-value < .05 was considered statistically significant.

### 2.9. Implementation details

The deep-learning and fusion models were implemented using Python 3.7 and PyTorch 1.11.0. Model training was performed on 2 NVIDIA RTX A4000 graphics cards. Each model was trained for 300 epochs with a batch size of 32. The initial learning rate was set to 0.0005, and the AdamW optimizer was used. Binary cross-entropy was adopted as the loss function. Radiomics feature extraction was conducted using PyRadiomics 1.0.1. All computations were performed on an Ubuntu 20.04 operating system with CUDA 11.3.

The code for the statistical analysis was made publicly available on the development platform GitHub at https://github.com/TangWen920812/EightModel4SuBei.

## 3. Results

### 3.1. The clinical characteristics of the patients

We retrospectively analyzed 353 patients with 387 lesions who met the inclusion criteria. Among these, 247 lesions were allocated to the training set, 62 to the validation set, and 78 to the test set.

Univariate analysis revealed significant differences in age, nodule diameter, and nodule attenuation between the growth and non-growth groups. No significant differences were observed in sex, smoking history, follow-up duration, or nodule location. Detailed clinical characteristics of the patients are summarized in Table 1.

**Table 1:** Clinical characteristics of the growth and non-growth groups in the training, validation, and test sets.

	Training set (*n* = 247)		*P*-value	Validation set (*n* = 62)		*P*-value	Test set (*n* = 78)		*P*-value
	Gg (*n* = 127)	Ng (*n* = 120)		Gg (*n* = 29)	Ng (*n* = 33)		Gg (*n* = 39)	Ng (*n* = 39)	
Age (yr)	59.13 ± 12.93	49.28 ± 13.28	<.05	57.17 ± 14.45	47.58 ± 14.23	<.05	57.56 ± 13.08	51.92 ± 15.66	.088
Gender*			.876			.399			.052
Male	70 (55.1%)	59 (49.2%)		17 (58.6%)	11 (33.3%)		18 (46.2%)	17 (43.6%)	
Female	57 (44.9%)	61 (50.8%)		12 (41.4%)	22 (66.7%)		21 (53.8%)	22 (56.4%)	
History of smoking	23 (18.1%)	25 (19.6%)	.292	5 (17.2%)	5 (15.1%)	.050	7 (17.9%)	9 (23.1%)	.315
Location*			.103			.278			.234
RUL	49 (38.6%)	45 (37.5%)		6 (20.7%)	10 (34.5%)		15 (38.5%)	13 (33.3%)	
RML	9 (7.1%)	2 (1.7%)		5 (17.2%)	2 (6.9%)		2 (5.1%)	4 (10.3%)	
RLL	20 (15.7%)	14 (11.7%)		7 (24.1%)	5 (17.2%)		8 (20.5%)	8 (20.5%)	
LUL	44 (34.6%)	36 (30.0%)		10 (34.5%)	11 (37.9%)		13 (33.3%)	8 (20.5%)	
LLL	14 (11.0%)	23 (19.2%)		1 (3.4%)	5 (17.2%)		1 (2.6%)	6 (15.4%)	
Diameter (mm)†	13.47 ± 5.22	7.82 ± 2.19	<.05	12.62 ± 8.78	7.64 ± 2.03	<.05	11.82 ± 4.90	7.67 ± 2.46	<.05
Attenuation			<.05			<.05			<.05
NSN	52 (40.9%)	88 (73.3%)		15 (51.7%)	26 (78.8%)		21 (53.8%)	33 (84.6%)	
PSN	75 (59.1%)	32 (26.7%)		14 (48.3%)	7 (21.2%)		18 (46.2%)	6 (15.4%)	
Follow-up period (mo)	36.83 ± 14.16	39.27 ± 11.00	.205	40.38 ± 17.85	40.55 ± 13.58	.944	37.38 ± 14.73	41.13 ± 12.63	.276

Gg = growth group, LLL = left lower lobe, LUL = left upper lobe, Ng = non-growth group, NSN = pure ground glass nodule, PSN = part solid nodule, RLL = right lower lobe, RML = right middle lobe, RUL = right upper lobe, SSN = subsolid nodule.

* Data are means ± standard deviations.

† Numbers in parentheses are percentages.

### 3.2. Radiomics analysis

**Figure 5.**
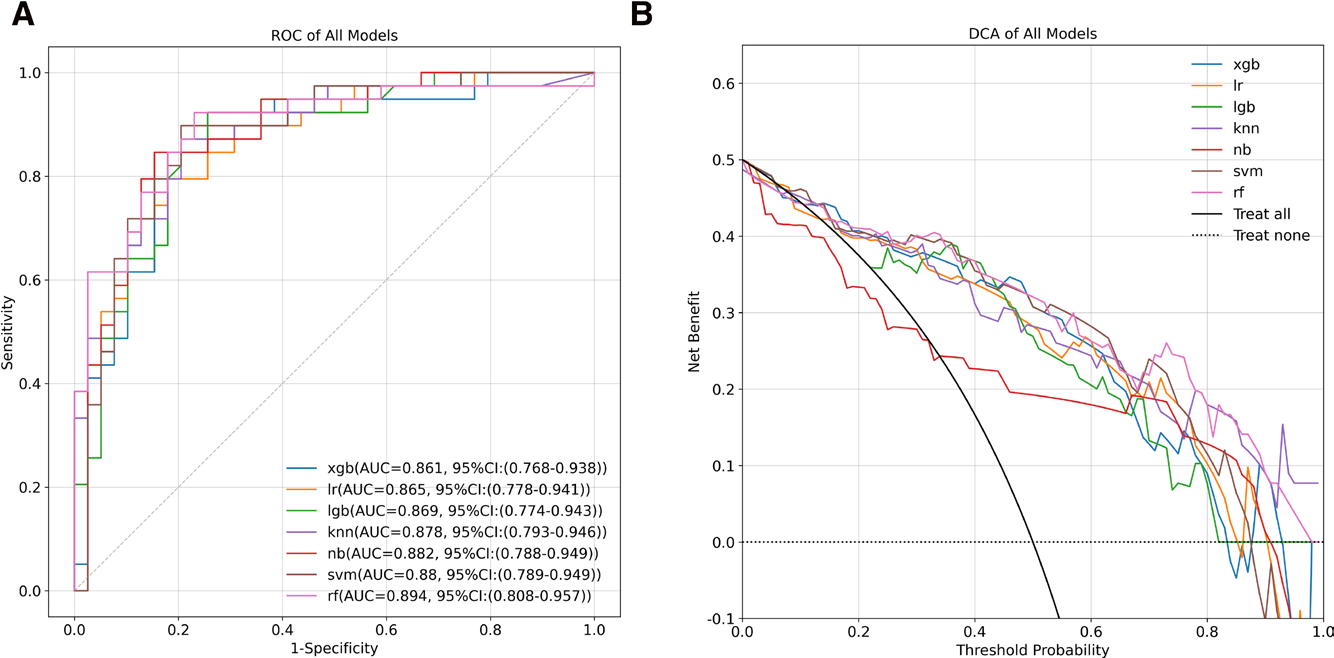
ROC curves (A) and DCA (B) of radiomics models based on 7 machine-learning algorithms in the test set. DCA = decision curve analysis, knn = k-nearest neighbor, lgb = LightGBM, lr = logistic regression, nb = Naïve Bayes, rf = random forest, ROC = receiver operating characteristic, svm = support vector machine, xgb = XGBoost.

A total of 30 radiomic features were selected, including first-order, GLCM, GLDM, GLSZM, NGTDM, and shape features. A radiomics model was developed to evaluate nodular progression using 7 machine-learning algorithms based on the selected features. The predictive performance of these models was assessed using CT-based prediction, with results from the test set summarized in Table 2. To further evaluate model performance, ROC curves and DCA for the 7 radiomics models were generated, as shown in Figure 5. Among the models, the Random Forest algorithm demonstrated the best performance, achieving an AUC of 0.894 (95% CI: 0.808–0.957), an accuracy of 0.846, a precision of 0.800, a recall of 0.923, and a specificity of 0.769 (see Supplemental Content 2 [Supplemental Digital Content, https://links.lww.com/MD/P754], which illustrates the confusion matrix of radiomics models based on 7 machine-learning algorithms in the test set).

**Table 2:** Predictive performance of 7 machine-learning methods based on radiomic features for subsolid nodule growth.

Model	AUC	95% CI	Accuracy	Sensitivity	Specificity	PPV	NPV	F1-score	AUPRC
xgb	0.861	0.768–0.938	0.833	0.846	0.821	0.825	0.842	0.835	0.833
lr	0.865	0.778–0.941	0.808	0.795	0.821	0.816	0.8	0.805	0.811
lgb	0.869	0.774–0.943	0.833	**0.923**	0.744	0.783	0.906	0.847	0.858
knn	0.878	0.793–0.946	0.833	0.872	0.795	0.81	0.861	0.84	0.887
nb	0.882	0.788–0.949	0.846	0.846	0.846	0.846	0.846	0.846	0.822
svm	0.88	0.789–0.949	0.846	0.897	0.795	0.814	0.886	0.854	0.817
rf	**0.894**	0.808–0.957	0.846	**0.923**	0.769	0.8	0.909	0.857	0.907

Bold values indicate the highest value.

AUC = area under the curve, AUPRC = area under precision recall curve, knn = k-nearest neighbor, lgb = LightGBM, lr = logistic regression, nb = Naïve Bayes, NPV = negative predictive value, PPV = positive predictive value, rf = random forest, svm = support vector machine, xgb = XGBoost.

### 3.3. Performance of the deep-learning model

A 2D ResNet18-based deep-learning model was developed to predict the progression of SSNs. The model achieved an AUC of 0.802 (95% CI: 0.695–0.899), with an accuracy of 0.756, sensitivity of 0.872, precision of 0.708, and specificity of 0.641 (see Supplemental Content 3 [Supplemental Digital Content, https://links.lww.com/MD/P754], which illustrates the confusion matrix the deep-learning model in the test set). Detailed results are summarized in Table 3.

**Table 3:** Predictive performance of radiomics, deep-learning and combined models for subsolid nodule growth.

Model	AUC	95% CI	Accuracy	Sensitivity	Specificity	PPV	NPV	F1-score	AUPRC
Radiomics	0.894	0.808–0.957	0.846	0.923	0.769	0.8	0.909	0.857	0.907
ResNet	0.802	0.695–0.899	0.756	0.872	0.641	0.708	0.833	0.782	0.828
ResNet + radiomics	**0.926**	0.869–0.977	**0.872**	0.846	**0.897**	0.892	0.854	0.868	0.926

Bold values indicate the highest value.

AUC = area under the curve, AUPRC = area under precision recall curve, NPV = negative predictive value, PPV = positive predictive value.

Representative Grad-CAM visualizations generated from the 2D ResNet18 model illustrate the regions that contributed most to the growth prediction (see Supplemental Content 4, Supplemental Digital Content, https://links.lww.com/MD/P754).

### 3.4. Combined radiomics and deep-learning models analysis

The combined model demonstrated superior performance compared to both the deep-learning model and the radiomic model, achieving the highest AUC of 0.926 (95% CI: 0.869–0.977). The combined model yielded an accuracy of 0.872, sensitivity of 0.846, precision of 0.892, and specificity of 0.897 (see Supplemental Content 5 [Supplemental Digital Content, https://links.lww.com/MD/P754], which illustrates the confusion matrix for the combined model in the test set).

**Figure 6.**
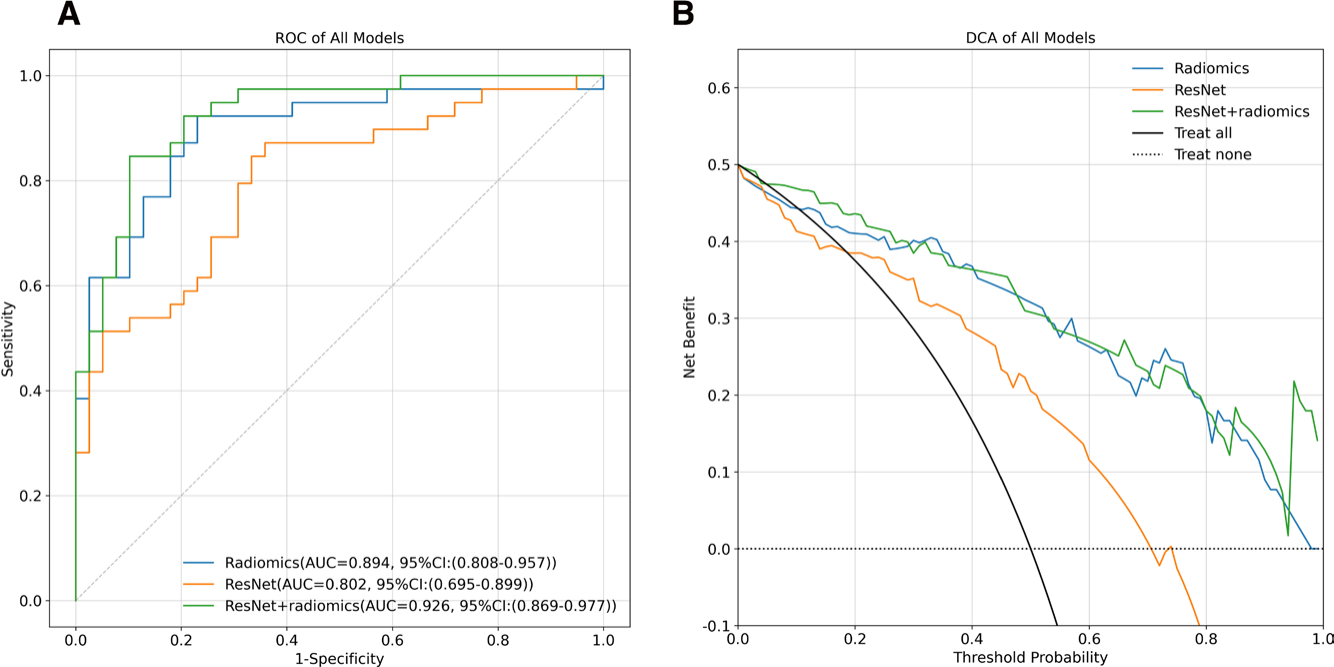
ROC curves (A) and decision curve analysis (B) of radiomics, deep-learning (ResNet) and combined (ResNet + radiomics) models in the test set. DCA = decision curve analysis, ROC = receiver operating characteristic.

**Figure 7.**
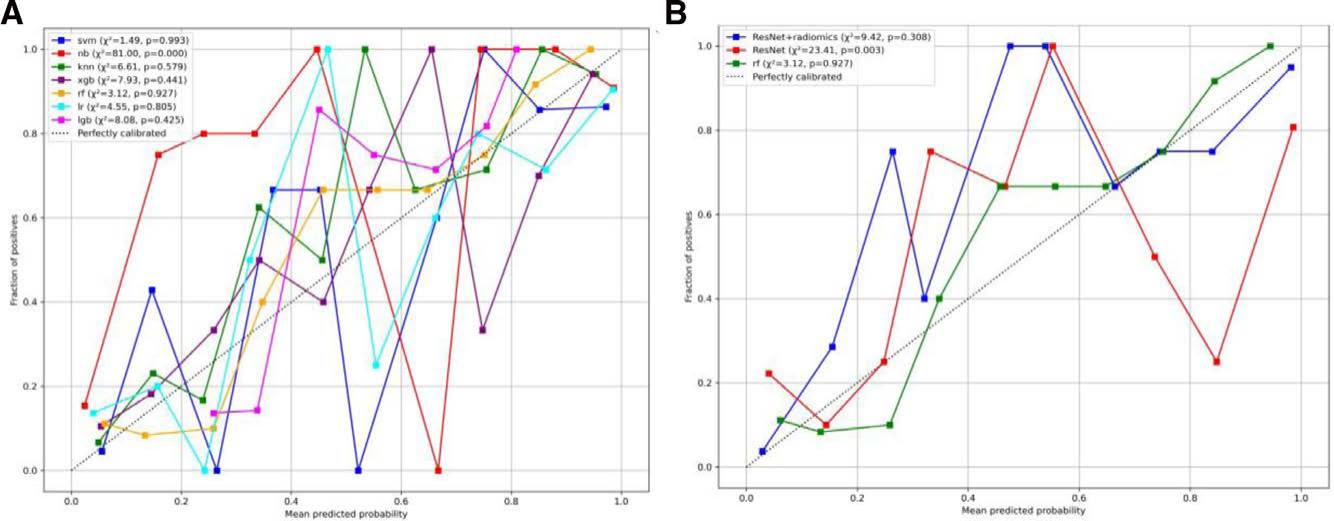
Calibration curves of radiomics, deep-learning (ResNet) and combined (ResNet + radiomics) models in the test set.

According to the Delong test, the differences between the radiomics model and the deep-learning model, as well as between the radiomics model and the combined model, were not statistically significant (*P* = .075 and *P* = .136, respectively). However, the difference between the combined model and the deep-learning model was statistically significant (*P* = .012). The performance metrics of the combined model, deep-learning model, and radiomics model are summarized in Table 3. The ROC and DCA for the combined model are shown in Figure 6. As shown in Figure 7, the Hosmer–Lemeshow test yielded *P*-values of .927, .003, and .308 for the radiomics model, the deep-learning model, and the combined model, respectively.

## 4. Discussion

The biological heterogeneity of SSNs presents substantial challenges in predicting the growth potential and selecting appropriate management strategies. Accurate assessment of SSN behavior is critical for risk stratification and the early diagnosis of lung cancer. In this study, we proposed a combined model that integrates radiomics and deep learning to predict SSN growth. The combined model achieved superior performance, with an AUC of 0.926 (95% CI: 0.869–0.977), outperforming the radiomics model (AUC = 0.894, 95% CI: 0.808–0.957) and the deep-learning model (AUC = 0.802, 95% CI: 0.695–0.899).

Previous studies have demonstrated the potential of radiomics in predicting the growth patterns of SSNs. For instance, Xue et al^[13]^ developed a radiomics-based nomogram to predict the growth of uncertain pulmonary nodules within 2 years. The nomogram achieved an AUC of 0.911, outperforming both the radiomics model (AUC = 0.892) and the clinical model (AUC = 0.812). Similarly, Tan et al^[14]^ constructed an imaging feature–radiomics model to predict the growth rate of 407 nodules, reporting that the combined model (AUC = 0.780) outperformed models based solely on imaging features (AUC = 0.727) or radiomics (AUC = 0.710). In another study, Zhou et al^[19]^ identified the radiomics characteristic model (RAD-score) and nodule diameter as independent predictors of SSN progression. The integrated clinical-radiomics model achieved an AUC of 0.801. These findings collectively underscore the utility of radiomics in SSN growth prediction. Consistent with prior work, our results suggest that even in relatively small-sample settings, radiomics can provide valuable insights into the natural evolution of SSNs and may facilitate more personalized risk stratification and follow-up strategies.

Radiomic features extracted from CT images offer quantitative imaging biomarkers that may reflect the underlying biological behavior of SSNs. First-order features (e.g., firstorder_10Percentile_wavelet-LHH, firstorder_Kurtosis_logarithm) characterize the intensity distribution and signal heterogeneity within the lesionn, which may reflect variations in tissue composition. Skewness or kurtosis may indicate non-uniform cell density, fibrosis, or early invasive components. Texture features derived from GLCM and GLDM (e.g., glcm_Correlation_wavelet-LLH, glcm_ClusterShade, gldm_DependenceVariance) indicate spatial dependencies and intralesional heterogeneity, which may reflect early architectural disruption, angiogenesis, or lepidic-to-invasive transitions. Features derived from GLSZM (e.g., glszm_SmallAreaLowGrayLevelEmphasis and SizeZoneNonUniformityNormalized), quantify the extent and uniformity of low-density regions, potentially corresponding to microinvasion, alveolar collapse, or scattered airspaces. In addition, shape-based features (e.g., Sphericity, SurfaceVolumeRatio, and Maximum2DDiameterColumn) describe the morphological complexity and boundary irregularity of nodules. Irregular or elongated shapes may be indicative of malignant transformation and enhanced invasive potential. Collectively, these radiomic descriptors serve as noninvasive imaging surrogates for the histopathological evolution of SSNs.

The innovation of our study lies in the integration of deep-learning representations with handcrafted radiomics features to characterize the phenotypic heterogeneity of SSNs. By leveraging both high-level semantic features from convolutional neural networks and quantitative texture and shape descriptors from radiomics, our combined model offers a more comprehensive understanding of SSN progression patterns. To the best of our knowledge, only a limited number of studies^[15,16]^ have systematically explored the prediction of SSN growth using deep-learning approaches. For example, Liao et al^[16]^ developed a deep-learning model based on a large dataset of 3120 SSNs. Their deep-learning model, termed “SiamModel” achieved superior performance compared to a radiomics-based model in the validation set, with an AUC of 0.858 (95% CI: 0.786–0.921) versus 0.760 (95% CI: 0.646–0.857), respectively. These findings support the potential of deep learning in capturing nuanced image patterns that may be overlooked by traditional radiomic features. Our study builds upon this foundation by not only validating the utility of deep learning but also demonstrating that its integration with radiomics can yield synergistic benefits. The ensemble model outperformed both individual approaches in terms of predictive accuracy and clinical utility, highlighting the value of multimodal learning strategies in medical image analysis.

In our study, the deep-learning model exhibited slightly inferior performance compared to the radiomics-based model. This contrasts with findings from Liao et al,^[16]^ where a deep learning approach outperformed conventional radiomics in predicting SSN growth. Several potential factors may explain this discrepancy. First, our model utilized 2D CT slices rather than full 3D volumetric inputs, potentially limiting its ability to capture the complete spatial context and volumetric heterogeneity of the nodules. Second, the relatively limited sample size in our dataset may have constrained the training efficiency and generalization ability of the deep neural network. Deep-learning models are known to require large-scale datasets to achieve optimal representation learning and model stability. In contrast, radiomics models, which rely on handcrafted and biologically interpretable features, are generally more robust in small-data scenarios. Additionally, differences in preprocessing pipelines, data augmentation strategies, and network architecture design may have further contributed to the observed performance gap. Notably, the calibration performance of the deep-learning model, as assessed by the Hosmer–Lemeshow test, was also suboptimal compared to the radiomics and fusion models. These findings underscore the critical importance of selecting appropriate modeling strategies, input formats, and calibration methods, particularly in small-sample medical imaging studies.

Although the deep-learning model may underperform when trained on limited datasets, integrating them with radiomics features presents a promising strategy. The fusion model benefits from the complementary strengths of both modalitie. Radiomics offers handcrafted, interpretable features rooted in domain knowledge, while deep learning captures high-dimensional, abstract representations from imaging data.^[20]^ This hybrid architecture enhances predictive accuracy, calibration, and robustness, while also improving the interpretability and clinical relevance. In our study, the combined model demonstrated superior performance over both individual models (AUC = 0.926) and showed acceptable calibration (*P* = .308), underscoring the added value of feature-level integration even in a small-sample setting.

Despite these encouraging results, our study has several limitations. First, the retrospective design may introduce selection or information bias. Second, the relatively small sample size may limit the generalizability and stability of the deep learning component, although the radiomics and fusion models still achieved satisfactory performance. Third, the model was developed and validated using data from a single center, which may restrict its applicability to broader populations or different imaging protocols. To address these limitations, future research will focus on prospective validation in a multicenter cohort, which will enable a more robust evaluation of model generalizability and clinical utility across diverse patient populations and imaging conditions.

In conclusion, we developed a high-performing predictive model that integrates radiomics and deep learning techniques for predicting SSN progression. This approach holds potential to support more accurate risk stratification, thereby informing personalized follow-up strategies and therapeutic decision-making in the early management of SSNs.

## Author contributions

**Conceptualization:** Dawei Wang, Kefu Liu.

**Data curation:** Wanying Yan, Xinyue Zhang.

**Formal analysis:** Jie Chen, Wanying Yan, Yiqiu Shi.

**Funding acquisition:** Jie Chen, Kefu Liu.

**Investigation:** Jie Chen, Yiqiu Shi.

**Methodology:** Wanying Yan, Lina Wang.

**Project administration:** Jie Chen.

**Resources:** Dawei Wang.

**Software:** Dawei Wang.

**Supervision:** Kefu Liu.

**Validation:** Ruize Yu.

**Visualization:** Wanying Yan, Ruize Yu.

**Writing – original draft:** Jie Chen, Wanying Yan, Xinyu Pan.

**Writing – review & editing:** Kefu Liu.

**Writing – review & editing:** Kefu Liu.

## Supplementary Material


