# Supplementary material for: Radiomics and deep learning methods for predicting the growth of subsolid nodules based on CT images
Source: Medicine (Baltimore). 2025 Aug 29;104(35):e44104. doi: 10.1097/MD.0000000000044104 (PMC12401382; doi:10.1097/MD.0000000000044104)
Supplement: Supplementary file 1 [file medi-104-e44104-s001.docx]

| Categories | Feature name |
| --- | --- |
| firstorder | firstorder_10Percentile_wavelet-LHH |
|  | firstorder_Energy_wavelet-LLH |
|  | firstorder_Kurtosis_logarithm |
|  | firstorder_Maximum_wavelet-LLH |
|  | firstorder_Mean_wavelet-LHH |
|  | firstorder_Median_wavelet-HHL |
|  | firstorder_Minimum_wavelet-HHH |
|  | firstorder_Minimum_wavelet-HLL |
|  | firstorder_Skewness_log-sigma-2-0-mm-3D |
| GLCM | glcm_ClusterShade_log-sigma-2-0-mm-3D |
|  | glcm_ClusterShade_wavelet-HHL |
|  | glcm_Correlation_wavelet-HHL |
|  | glcm_Correlation_wavelet-HLL |
|  | glcm_Correlation_wavelet-LHL |
|  | glcm_Correlation_wavelet-LLH |
|  | glcm_Imc1_wavelet-LHH |
|  | glcm_Imc2_wavelet-HHL |
| GLDM | gldm_DependenceVariance_wavelet-HHH |
|  | gldm_LowGrayLevelEmphasis_wavelet-LLL |
| GLSZM | glszm_SizeZoneNonUniformityNormalized_square |
|  | glszm_SizeZoneNonUniformityNormalized_wavelet-HLL |
|  | glszm_SmallAreaEmphasis_log-sigma-3-0-mm-3D |
|  | glszm_SmallAreaLowGrayLevelEmphasis_log-sigma-3-0-mm-3D |
| NGTDM | ngtdm_Busyness_wavelet-LLH |
|  | ngtdm_Contrast_wavelet-HHH |
|  | ngtdm_Strength_square |
|  | ngtdm_Strength_wavelet-HHH |
| shape | shape_Maximum2DDiameterColumn_original |
|  | shape_Sphericity_original |
|  | shape_SurfaceVolumeRatio_original |

**Supplemental Digital Content 1.** Table that illustrates the radiomics features retained after feature selection.

A
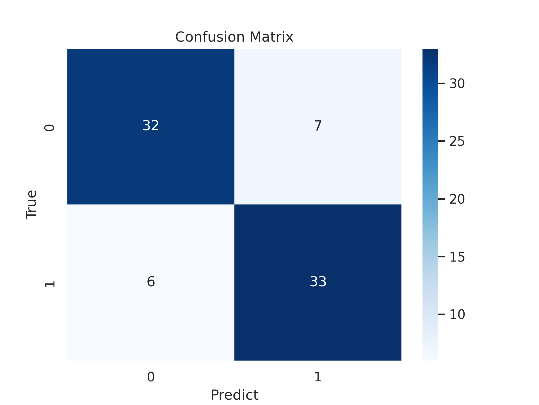
 B
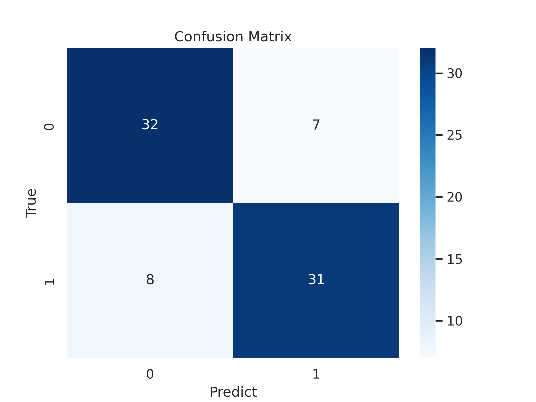


C
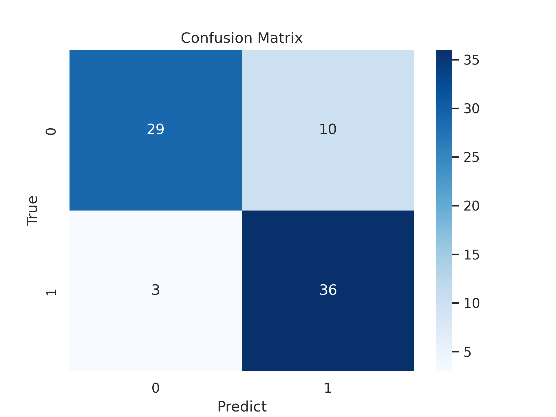
 D
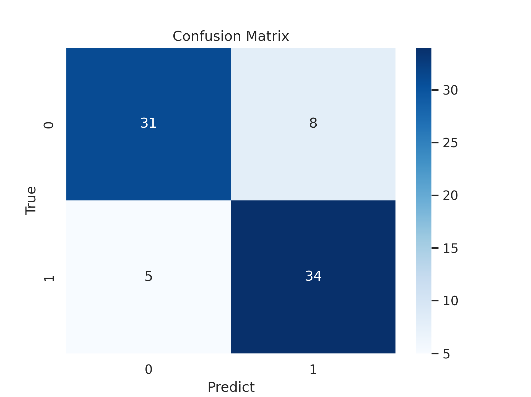


E
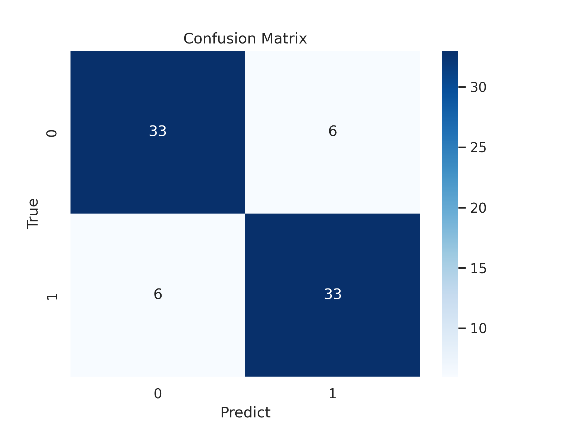
 F
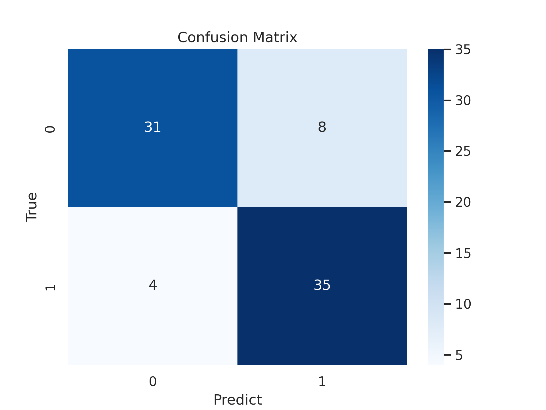


G
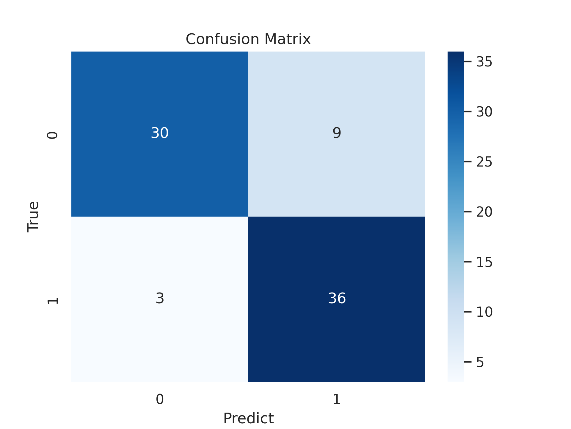


**Supplemental Digital Content 2.** Figure that illustrates the confusion matrix of radiomics models based on seven machine-learning algorithms in the test set. (A.XGBoost; B.Logistic Regression; C. LightGBM; D. K-Nearest Neighbor; E. Naïve Bayes; F.Support Vector Machine; G.Random Forest)


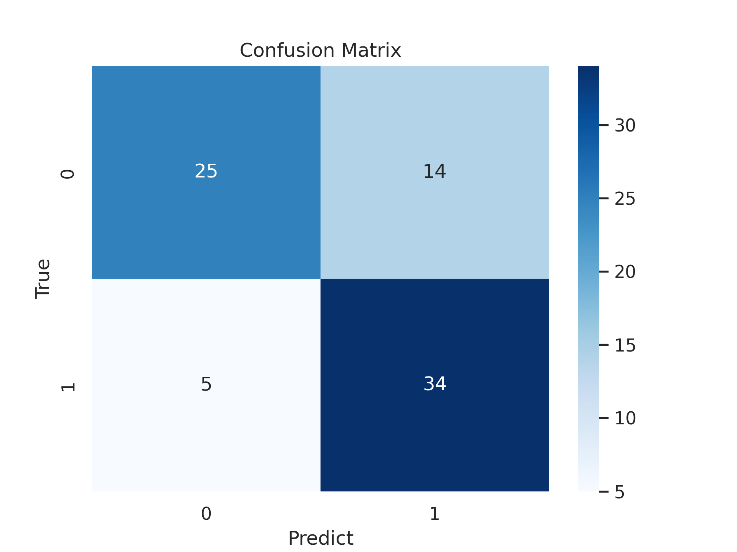


**Supplemental Digital Content 3.** Figure that illustrates the confusion matrix of the deep-learning model in the test set.


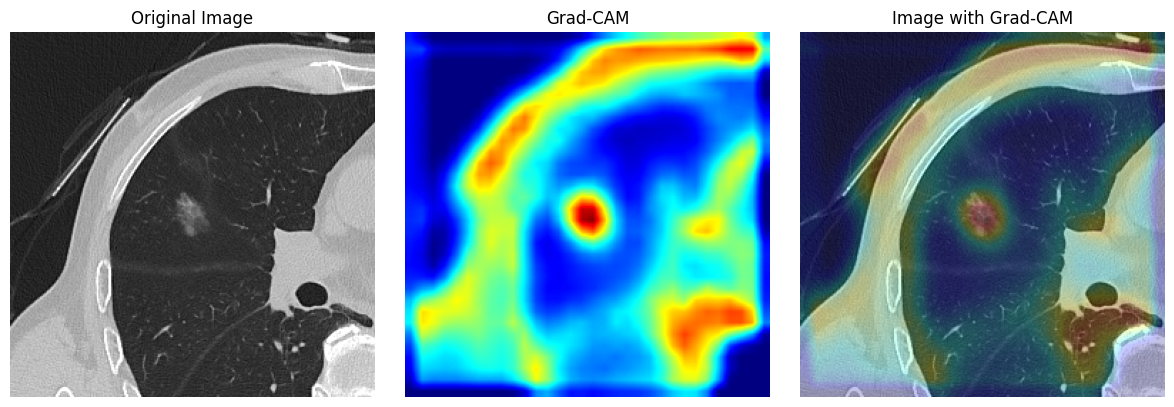


**Supplemental Digital Content 4.** Figure that illustrates the Grad-CAM visualizations for the deep-learning model in the test set.


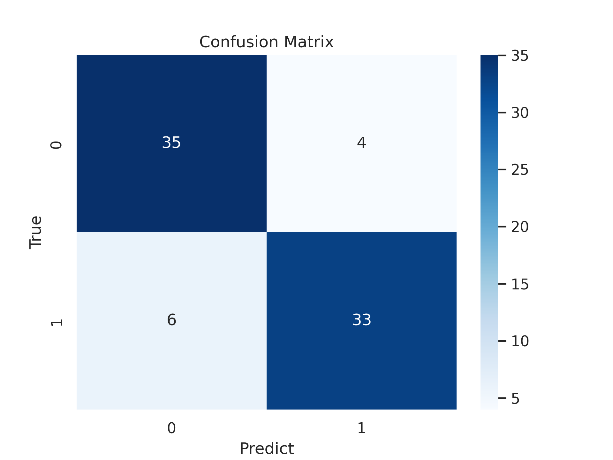


**Supplemental Digital Content 5.** Figure that illustrates the confusion matrix of the combined model (radiomics+deep-learning) in the test set.
